# Assessing eating disorder education in U.S. medical schools: a qualitative content analysis of lecture slides

**DOI:** 10.1007/s40519-025-01799-0

**Published:** 2025-11-28

**Authors:** Agatha A. Laboe, Lauren E. Pictor, Mahathi Gavuji, Samantha Temucin, Anna Kreynin, Elizabeth Sheil, Brooke Jourdan, Katherine Schaumberg, Heather Davis, Erin N. Harrop

**Affiliations:** 1 Department of Psychology, The University of Texas at Austin, Austin, TX USA; 2 Department of Psychological Sciences, Auburn University, Auburn, AL USA; 3 Philadelphia College of Osteopathic Medicine, Philadelphia, PA USA; 4 Department of Psychiatry, University of Wisconsin-Madison, Madison, WI USA; 5 Department of Psychology, University of Wisconsin-Madison, Madison, WI USA; 6 Department of Psychology, Virginia Polytechnic Institute and State University, and affiliate faculty at the Virginia Tech Child Study Center, Blacksburg, VA USA; 7 School of Social Work, University of Denver, Denver, CO USA

**Keywords:** Medical education, Eating disorders, Weight stigma, Physician, Early detection

## Abstract

**Purpose:**

Physicians can play a critical role in the early identification and treatment of eating disorders (EDs), yet many report low confidence in diagnosing and managing these illnesses. ED education during medical training has the potential to improve physician’s competence in recognizing, diagnosing, and treating EDs. This study assessed the content of ED education in preclinical medical training to identify opportunities to strengthen curricula.

**Methods:**

Slide content from 15 ED lecture presentations used between 2019 and 2024 in preclinical medical education at either Doctor of Medicine (MD) or Doctor of Osteopathic Medicine (DO) institutions in the United States was analyzed. Through a directed qualitative content analysis, the depth and breadth of textual content were examined and the use of visual aids in slide content was assessed.

**Results:**

Slides often acknowledged the multifactorial nature of EDs. They primarily focused on anorexia nervosa and bulimia nervosa, but not other EDs. Images reinforced stereotypes about who develops EDs, and there was limited mention of weight stigma as a barrier to diagnosis and treatment. Furthermore, ED treatment options, including therapeutic modalities, pharmacotherapies, and levels of care, were introduced but not described in detail.

**Conclusion:**

Physicians are well-positioned to identify and treat EDs. However, findings suggest that significant gaps exist in the coverage of EDs in preclinical medical education. Current preclinical education may inadvertently reinforce misconceptions that hinder the ability of physicians to detect diverse presentations of EDs early. Recommendations are offered to guide future lecture development and strengthen ED-related educational content.

**Level of evidence:**

Level V: Opinions of respected authorities, based on descriptive studies, narrative reviews, clinical experience, or reports of expert committees.

## Introduction

Eating disorders (EDs) are severe, potentially life-threatening health conditions that affect nearly 9% of the US population over their lifetime [1]. Early detection and timely access to ED treatment are imperative for improving prognosis [2–4]. Prior to referral to specialty mental health care, individuals with EDs may present in different medical settings, including primary care, gastroenterology, obstetrics/gynecology, and emergency departments, with or without complaints related to ED symptoms [5–7]. This pattern creates a critical opportunity for physicians to screen for EDs, conduct essential medical tests, and connect patients with specialized care before the disorder progresses [8].

### Lack of comfort and weight stigma may hinder ED care

Despite the potential for physicians to detect EDs early, they report low comfort and confidence with managing EDs [8–11]. In a survey of 80 resident physicians and fellows across disciplines, only 11.4% expressed comfort working with patients with EDs [9]. Another survey of 92 medical residents in internal medicine, emergency medicine, obstetrics/gynecology, psychiatry, and surgery found that only a minority felt confident diagnosing (28%), treating (17.4%), or discussing (30%) disordered eating with patients, and most respondents (69%) reported a lack of knowledge regarding EDs among sexual, gender, racial or ethnic minorities [10]. Overall, widespread discomfort and lack of confidence among physicians may be barriers to early detection and intervention for patients with EDs.

Beyond discomfort and lack of confidence, practitioner’s biases towards EDs may further contribute to underdetection and misdiagnosis, particularly for patients at higher weights. Narrative data from patients with EDs highlights that healthcare providers often pathologize high body weight and in some cases, praise disordered eating behaviors in individuals at higher weights [12]. This weight stigma contributes to disparities in ED identification and clinical decision-making. For example, in cases of atypical anorexia nervosa (atypical AN, in which patients have “normal” or higher body mass index), physicians are not only less likely to provide a diagnosis, compared to low-weight anorexia nervosa (AN) [13], but they are also less likely to recommend follow-ups, refer to specialists, or have a structured treatment approach [14], despite similar presentations, symptoms, behaviors, and medical consequences [15, 16] and atypical AN being more prevalent [17]. Binge eating disorder (BED) is also frequently overlooked, in part because clinicians may attribute weight concerns to lifestyle factors rather than considering BED [18]. Compounding these issues, medical trainees report witnessing weight stigma in clinical settings [19, 20]. While it is unclear how much weight stigma among physicians results from medical training versus larger cultural biases, these biases likely interfere with early detection and treatment of EDs.

### ED-related training in medical education

Importantly, ED-focused didactic and clinical trainings have been associated with greater comfort and confidence in diagnosing and treating EDs and may also reduce bias [9, 21, 22]. Ensuring that medical students receive adequate ED training, therefore, is a crucial step toward enhancing early detection and intervention in medical settings. Notably, U.S. medical education follows a spiral curriculum, whereby training unfolds across multiple stages: concepts are introduced during the preclinical years, applied and reinforced during clinical clerkships, and developed into specialty-specific expertise during residency and beyond. This structure provides multiple, scaffolded opportunities for ED-related training.

To date, research on ED-related training throughout medical education has focused on the residency level. In a study of 637 medical residency programs across internal medicine, pediatrics, family medicine, psychiatry, and child and adolescent psychiatry, 81% did not offer any scheduled or elective rotations focused on EDs, and of the 123 programs that did, fewer than half included a formal, scheduled rotation [23]. Similarly, a survey of 880 medical residents in family medicine, pediatrics, psychiatry, internal medicine, emergency medicine, and obstetrics/gynecology found that 70% had received fewer than five hours of training on child and adolescent EDs, and only 24% believed their medical education had adequately prepared them to address EDs [21]. These findings underscore that training gaps in EDs exist at advanced stages of medical education.

However, less is known about how EDs are introduced in the preclinical years, a foundational stage during which exposure to ED content may influence whether students feel prepared to recognize and manage these disorders in clinical practice, as well as whether they pursue further training in this area. This stage is also critical for mitigating biases and stereotypes related to EDs. Importantly, what students learn in this period may be influenced by structural forces such as the United States Medical Licensing Examination (USMLE), a three-part exam series that all medical students must pass to practice in the U.S. [24]. For example, the USMLE content outline lists only AN, bulimia nervosa (BN), and binge eating disorder (BED), which may limit classroom teaching and external resources (e.g., First Aid, Anki), while shaping student’s perceptions of which disorders are clinically relevant. Due to time constraints, medical students increasingly utilize external study resources in preparation for the USMLE examinations, citing their conciseness and ability to capture attention [25, 26]. The increasing use of external resources may highlight the need for preclinical curriculum to intentionally recognize and mitigate any bias these resources may be introducing.

### Current study

Against this backdrop, the present study examines how EDs are introduced in preclinical medical education, a foundational stage for future clinical practice, by analyzing lecture slide content from U.S. medical schools. Using directed qualitative content analysis—an analytic approach that applies prior research to guide coding and interpretation of data and generates descriptive accounts of the material under study [27]—we (1) examined the depth and breadth of the textual content in lecture slides and (2) assessed the use of visual aids. This approach provided a direct assessment of what preclinical medical students are taught in the classroom about EDs. By highlighting both effective practices and areas for improvement, results inform concrete recommendations to improve ED training in preclinical medical education, equipping future physicians to identify EDs early and facilitate timely referrals to appropriate care.

## Method

### Lecture presentations

Lecture slides were analyzed for the present study, as medical educators rely heavily on lecture slides, and medical students report using them to study [28]. Inclusion criteria required that lecture slides had been used in 2019–2024 in a United States Doctor of Medicine (MD) or Doctor of Osteopathic Medicine (DO) program. Additionally, approval from the course instructor was required for the slides to be included. We gathered slides by posting on social media, emailing individuals in leadership positions at all U.S. medical schools (i.e., Directors of Medical Education and Curriculum, Associate Deans for Medical Education, Curriculum Coordinators, or Chairs of Psychiatry, depending on publicly available contact information), sharing information about the study through medical student listservs (e.g., Medical Students for Size Inclusivity), and contacting medical students with whom we had personal connections. This study was waived by the University of Wisconsin-Madison IRB because human subjects data were not used.

### Analytic strategy: directed qualitative content analysis

Directed qualitative content analysis is a combined deductive and inductive approach. It begins with a structured coding framework based on existing research, which is then refined through iterative data analysis. Researchers then identify themes by grouping and interpreting patterns in the codes [27]. Full details on how we employed directed qualitative content analysis for the current project are provided below.

Prior to analysis, our authorship team engaged in bracketing by reflecting on our positionality and preconceived ideas about medical school ED education to increase our awareness of personal assumptions that might impact data interpretation. Example assumptions included: (a) medical schools do not teach extensively about other specified feeding and eating disorders, including atypical anorexia nervosa and (b) curriculum on binge eating disorder is based on weight-centric views. Members of the authorship team have disciplinary backgrounds in medicine, psychology, and social work. Multiple individuals have lived experience with an ED and/or lived experience working with patients with EDs. Our team included the following identities: racial identity—White (9/10); body size—larger-bodied (2/10); gender identity—nonbinary (2/10) and cisgender woman (8/10); and highest degree attained—high school (1/10), bachelor’s (4/10), master’s (2/10), and doctorate (3/10).

To develop the text analysis and visual analysis coding guides, we reviewed the first eight lecture presentations and generated potential codes. These codes were inductive (i.e., arising from the data) and were combined with a list of a priori, deductive codes (i.e., grounded in previous literature and theory) developed during study conceptualization. Lecture presentations were test-coded, and coding guides were updated accordingly. The final coding guides (available from the first author upon request) were discussed and approved by all members of the study team.

Lecture slides were analyzed by six coders (A.A.L., M.G., S.T., A.K., L.E.P., E.S.) using established coding conventions, with coding discrepancies resolved through consensus. To enhance the rigor of our analysis, we counted the frequency of codes to help us identify recurring patterns and substantiate the presence of certain concepts across lecture presentations. Code counting was conducted systematically, with counts independently verified by two coders to ensure accuracy.

After all lecture presentations were coded with the final set of codes, the coding team grouped codes into subthemes based on their interconnected context within the presentations. Then, subthemes were grouped into overall themes.

## Results

### Descriptive statistics

Lecture presentations from 15 medical schools were used in this study. Most were from MD programs (*n* = 11, 73.3%), compared to DO programs (*n* = 4, 26.6%). Schools were in various geographic areas across the U.S, including the Midwest (*n* = 6, 40.0%), Northeast (*n* = 5, 33.3%), Southeast, (*n* = 3, 20.0%) and West (*n* = 1, 6.7%). The majority (*n* = 9, 60.0%) were public. See Table 1 for more details on medical school characteristics. This sample is reflective of the overall distribution of medical schools in the United States [29, 30].

**Table 1:** Medical school descriptives

Variable	*n*(%)
MD vs DO	
MD	12 (80.0%)
DO	3 (20.0%)
Geographic region	
Midwest	6 (40.0%)
Northeast	5 (33.3%)
Southeast	3 (20.0%)
West	1 (6.7%)
Public vs private	
Public	9 (60.0%)
Private	6 (40%)
US news and world report ranking: best medical schools—research	
Unranked	9 (60.0%)
Tier 3	3 (20.0%)
Tier 2	2 (13.3%)
Tier 1	1 (6.7%)

Fifteen lecture presentations were included in the text analysis (711 total slides). On average, lecture presentations had 47.4 slides (*SD* = 38.21, range = 4–159). For the visual analysis, 13 lecture presentations included images (193 total images). On average, lecture presentations that included images had 14.9 images (*SD* = 10, range = 3–35). The duration of the lectures, verbal presentation of material not explicitly written in slide content, and whether additional materials or discussions on EDs were included beyond these lectures within the broader curriculum are unknown.

### Qualitative results

**Fig. 1:**
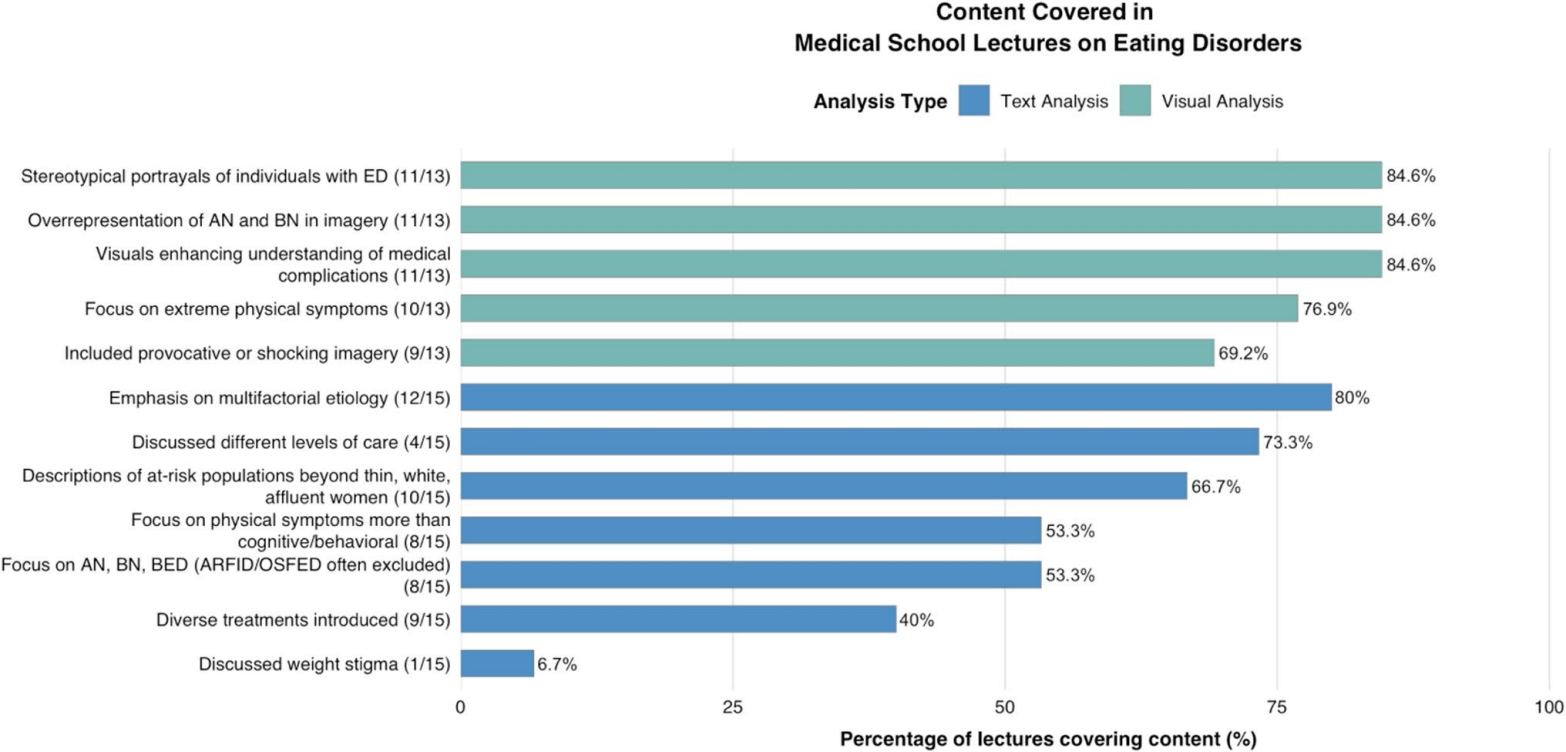
Content Covered In Medical School Lectures on Eating Disorders *ED* eating disorder, *AN* anorexia nervosa, *BN* bulimia nervosa, *BED* binge eating disorder, *ARFID* avoidant-restrictive food intake disorder, *OSFED* otherwise specified feeding and eating disorder. This figure depicts how often different content was covered in lecture slides, differing slightly from the themes identified in the analysis

Results are summarized in Fig. 1

#### Text analysis

The following analysis explores the themes identified in the analysis of the depth and breadth of the textual content in 15 ED lecture presentations. Identified themes were inductively organized into three overarching categories based on their focus within lecture content: (1) epidemiology and risk factors, (2) ED diagnosis and symptomatology, and (3) ED treatment.

#### Epidemiology and risk factors

Twelve (80%) lecture presentations emphasized the multifactorial etiology of EDs; 3 (20%) did not mention any risk factors. Twelve (80%) identified family history/genetics as risk factors, including statistics such as “EDs have a heritability between 30 and 80%”, and EDs exhibit “higher rates in monozygotic twins.” Twelve (80%) also named sociocultural factors, such as belonging to “interest groups” including “dance, bodybuilding, modeling, wrestling, cross country runners, gymnastics, figure skating, and acting”; “crisis with the family, school, relationship, or sexuality”; and “exposure to media” as contributors to EDs. Twelve (80%) additionally mentioned dieting as a risk factor. A large proportion (*n* = 11, 73.3%) highlighted temperament/personality traits, such as “perfectionism, compulsivity, anxiety, insecurity, rigidity” as risk factors.

Nearly three-quarters (*n* = 11, 73.3%) of slide decks described at-risk groups for EDs, and descriptions varied in their comprehensiveness. Specific foci of analysis included the degree to which EDs were discussed as presenting across body size, race/ethnicity, socioeconomic status, and gender.

*Body size.* Almost half (*n* = 6, 40%) highlighted that people of all body sizes can experience an ED, often in the context of bulimia nervosa (BN) or binge eating disorder (BED). For example, one slide describing the prevalence and course of BED noted that it occurs in “2.9% of overweight individuals” and is “more common in patients seeking weight loss treatment.”

*Racial/ethnic groups.* Over half (*n* = 8, 53.3%) included information about EDs across different racial groups, most frequently in the context of BN or BED. For example, one slide covering the epidemiology of BN and BED stated, “BN also affects women of color”, and “BED occurs in all racial groups.”

*Socioeconomic status.* Six (40%) presentations discussed EDs across socioeconomic statuses (SES), with some noting that EDs disproportionately occur among “higher SES” individuals and others highlighting that EDs occur across “all socioeconomic groups.”

*Gender.* In ten (66.7%) presentations, EDs were acknowledged as affecting individuals of all genders, most commonly through male-to-female prevalence ratios. Some presentations also specifically addressed EDs in men, transgender, and nonbinary populations. Three (20%) presentations explicitly refuted the idea that EDs solely affect thin, white, affluent women. For example, one slide emphasized, “Although EDs historically have been reported as more common in females than males, males may be under-identified. EDs also occur in culturally, ethnically, and socioeconomically diverse populations. It is important for clinicians to remain vigilant for possible ED symptoms regardless of patient demographics.”

Overall, there was a notable absence of discussion of weight, racial and ethnic diversity, and SES diversity among those with AN-spectrum symptoms.

#### ED diagnosis and symptomatology

In eight (53.3%) lecture presentations, AN, BN, and BED were emphasized, with avoidant-restrictive food intake disorder (ARFID) and otherwise specified feeding and eating disorder (OSFED) not mentioned. AN and BN were included in all presentations, while BED appeared in 13 (86.7%). Regarding OSFED subtypes, atypical AN was mentioned in four (26.7%) lectures, and purging disorder was mentioned in five (33.3%). Notably, when ARFID or OSFED were included, their coverage was scant relative to the coverage of AN and BN, and often limited to a review of the DSM-5 criteria.

Slides also focused on physical symptoms of EDs relative to cognitive and behavioral symptoms. Over half (*n* = 8, 53.3%) of presentations covered more physical symptoms than cognitive or behavioral symptoms. All presentations included physical symptoms, such as “low bone density”, “lanugo”, and “dental erosion”. The majority (*n* = 12, 80%) included cognitive symptoms, such as “weight phobia” and “cognitive rigidity” and behavioral symptoms, such as “social withdrawal”. Notably, physical symptoms were predominantly presented in the context of AN or BN.

There was limited mention of weight stigma’s role in hindering ED diagnosis and treatment. The vast majority (*n* = 14, 93.3%) of lecture presentations did *not* mention weight stigma. This presentation defined weight stigma as “the larger systemic discriminatory practices targeted at someone because of weight/body size” and incorporated a section on “re-examining the role of the scale”, encouraging students to reflect on the messages medical professionals send by focusing on weight.

#### ED treatment

Twelve (80%) presentations introduced treatments for EDs, including nutritional restoration, pharmacotherapy, and specific psychotherapy models (e.g., Family-Based Treatment). Of those that covered treatment, three (25%) described treatment for EDs in detail. For example, although 80% (*n* = 12) of presentations mentioned nutritional restoration, it was frequently in the context of AN only, and 33.3% (*n* = 4) of those mentioning nutritional restoration covered refeeding syndrome. Pharmacotherapy and psychotherapy models were also frequently referred to by name but rarely described.

Nearly three-quarters (*n* = 11, 73.3%) of lecture presentations did not reference different levels of ED care. Among the four (26.7%) that did, discussions were often limited to listing the levels of care without providing guidance on how to determine appropriate referrals. Outpatient care was mentioned in seven (46.7%) presentations, while intensive outpatient, partial hospitalization, and residential care were each referenced in only two (13.3%). Inpatient care was the most frequently discussed (*n* = 8, 53.3%), with the greatest detail focused on criteria for inpatient referrals. For example, “heart rate < 40 bpm, blood pressure < 90/60 mm Hg, symptomatic hypoglycemia, and potassium < 3 mmol per liter” were highlighted as specific criteria. Inpatient treatment was almost solely presented in the context of treatment for AN.

#### Visual analysis

The following analysis explores the themes identified in the analysis of the use of visual aids in 13 ED lecture presentations. Identified themes were inductively organized into two overarching categories based on their content: (1) ED symptomatology and (2) ED portrayal.

#### ED symptomatology

Most presentations included more images of severe, late-stage physical symptoms (*n* = 10, 76.9%), such as lanugo (*n* = 5, 38.5%), hair loss (*n* = 3, 23.1%), Russell’s sign (*n* = 5, 38.5%), and low bone density (*n* = 3, 23.1%), compared to earlier physical warning signs such as dry skin (*n* = 1, 7.7%) and menstrual irregularity (*n* = 2, 15.4%).

In 84.6% (*n* = 11) of presentations, medical complications of EDs were visually depicted. Over half (*n* = 7, 53.8%) of presentations included diagrams of how EDs—particularly AN and/or BN—affect the body. Nine (69.2%) included visual representations of medical complications, such as images of the different organ systems to highlight how all systems are implicated during an ED.

#### ED portrayal

AN and BN were more frequently represented by images compared to other EDs. The majority (*n* = 11, 84.6%) of lecture presentations included representations of both AN and BN, whereas five (38.5%) represented BED, nine (69.2%) represented ARFID, and one (7.7%) represented OSFED. For AN, images often depicted individuals presumed to have AN based on body appearance. For BN and BED, there was a mix of individuals—particularly celebrities—presumed to have these EDs and symbolic representations of EDs through images of highly palatable foods (e.g., chocolate chip cookies, chips). For ARFID, there were primarily graphics illustrating ARFID subtypes.

Images of individuals presumed to have EDs in lecture presentations largely reinforced the stereotype that EDs primarily affect thin, white young women. Specifically, 76.9% (*n* = 10) featured a teenager/young adult, 92.3% (*n* = 12) depicted a very thin individual, 100% (*n* = 13) included a feminine-presenting individual, and 100% (*n* = 13) portrayed a white-passing individual. In contrast, representation of diverse identities was limited, with zero (0.0%) presentations including older adults, zero (0.0%) depicting a larger-bodied individual, 13 (46.2%) featuring a non-feminine-presenting individual, and five (38.5%) including a non-white-passing individual.

Images of individuals presumed to have EDs were often sensationalized and extreme. Eight (61.5%) lecture presentations included an image featuring individuals in revealing clothing and nine (69.2%) depicted emaciated individuals with visibly protruding bones.

## Discussion

Our analysis of lecture slides from 15 US medical schools revealed how EDs are described within preclinical medical education. While lectures conveyed that EDs result from multiple interacting factors, they primarily emphasized physical symptoms and focused disproportionately on low-weight AN and BN. Additionally, slide content reinforced stereotypes about who develops EDs, particularly through visuals. Weight stigma was rarely mentioned as a barrier to ED identification and treatment, and no higher weight individuals were featured in images. Finally, treatment details provided little guidance on therapeutic approaches and levels of care. Overall, findings suggest that current preclinical education may inadvertently reinforce misconceptions about EDs that hinder early detection and treatment referral, particularly among patient groups already underrepresented in treatment settings (e.g., males, gender diverse individuals, older adults, individuals of diverse ethnic and racial backgrounds, individuals at higher weights, individuals of low SES).

### Interpreting these findings in the context of preclinical medical education

As described below, based on our findings, there are multiple opportunities to improve ED training in the preclinical years. However, our findings must be considered within the goals and constraints of preclinical education. Because the preclinical curriculum must cover a vast array of topics, educators may face time constraints and prioritize content that aligns with high-stakes exams such as the USMLE [24]. Medical education generally follows a spiral curriculum in which topics are revisited during the clinical years. However, preclinical education often represents the only guaranteed opportunity for exposure to certain subjects, as clinical experiences can vary substantially. As such, structural factors may help explain the limited depth and scope of ED coverage, but it is imperative to ensure that foundational teaching avoids perpetuating misconceptions and adequately prepares students to recognize EDs in diverse patient populations.

### Medicalization of EDs

Turning to our findings, the medicalization of EDs was evident in the strong focus on physical symptoms (through text) and medical complications (through visuals). The emphasis on physical symptoms and medical complications aligns with the focus placed on them in the UMSLE [24], but may shape future clinician’s diagnostic heuristics, leading them to associate EDs primarily with severe medical consequences rather than earlier, subtler symptoms. The physical symptoms highlighted in lecture slides were often late-stage symptoms of primarily restrictive EDs, such as lanugo or low bone density, rather than earlier warning signs including fatigue, dry skin, menstrual irregularity, and sore throat [31]. This narrow framing risks delays in diagnosis and intervention, as physicians may not recognize the need to screen patients who do not yet exhibit severe physical deterioration. Teaching students to include EDs in their list of differential diagnoses when patients present with vague physical (e.g., constipation, anemia, dizziness) or cognitive (e.g., poor concentration, insomnia) symptoms may decrease the delay in diagnosis and intervention.

### Overrepresentation of low-weight AN and BN

Lecture content overwhelmingly emphasized low-weight AN and BN, aligning with previous findings that medical trainees feel most comfortable assessing and treating low-weight AN, compared to other EDs [21]. In contrast, BED, ARFID, and OSFED were often absent or only briefly mentioned. This imbalance has real-world implications, particularly given that BED and OSFED have higher lifetime prevalence (BED: up to 6.1% of women and 0.7% of men; OSFED: up to 11.5% of women and 4.7% of men) than AN (up to 6.3% of women and 0.3% of men) or BN (up to 2.6% of women and 0.2% of men) [32]. Additionally, like AN and BN, BED, ARFID, and OSFED can be psychologically and physically debilitating [33], further underscoring their need for inclusion.

In terms of depiction of EDs, the lack of representation of higher weight bodies in content regarding AN symptoms and presentations could contribute to underdiagnosis and undertreatment of atypical AN compared to AN [13, 14]. Previous literature suggests that the preponderance of low-weight case examples used within AN clinical vignettes in medical education may contribute to underrecognition of starvation in non-emaciated patients [34]. Ultimately, without coverage of all EDs, preclinical medical education may inadvertently reinforce diagnostic and treatment disparities.

Notably, structural factors may be particularly salient to the disproportionate focus on low-weight AN and BN. For example, only AN, BN, and BED are mentioned in the content outline for the USMLE [24], which may limit incentives for schools to cover other EDs. Furthermore, ED research is rapidly evolving—for instance, most studies on atypical AN have been published within the past 5 years—making it difficult for lecturers to keep materials current, especially if they are not content experts. Future efforts could focus on the co-development of lecture materials with medical school faculty and ED researchers to ensure more contemporary coverage. Recognizing that our findings on the current state of preclinical education suggests gaps in ED education (e.g., those receiving preclinical training even 5–10 years ago may not have a robust didactic understanding of emerging areas of ED research including atypical AN, BED, or OSFED), ongoing education for resident physicians and current practitioners is also important. This is particularly relevant as attending physicians are often primary clinical supervisors, supporting ongoing knowledge development, during the clinical training years.

### Limited mention of weight stigma

Despite its pervasive presence in health care settings [19, 20] and its documented role as a risk factor for the development and maintenance of EDs [35], weight stigma was only acknowledged in one presentation. Given that weight stigma among medical professionals can lead to misdiagnosis, stigma-driven treatment disparities, and avoidance of healthcare among patients [13], this omission represents a gap in current preclinical medical training on EDs. For example, weight stigma may contribute to the underdiagnosis of EDs in individuals in higher-weight bodies, despite the presence of severe symptoms [13, 14]. Without recognizing weight stigma as a barrier to ED diagnosis and overall healthcare, future physicians may unintentionally perpetuate it, undermining their ability to provide effective ED intervention.

### Reinforcement of ED stereotypes

The visual content in ED lectures perpetuated misleading stereotypes of who develops EDs. Many images depicted extremely thin white women, particularly in representations of AN. Past research indicates physicians are more likely to diagnose EDs in women than in men, even when clinical presentations are identical [36]. Additionally, medical trainees often report feeling unprepared to assess and treat EDs in patients from minoritized racial, ethnic, gender, and socioeconomic backgrounds [10]. The limited diversity in ED representations may reinforce biases that contribute to disparities in diagnosis and treatment access. Future training should explicitly challenge these stereotypes by emphasizing that EDs occur across all genders, body sizes, racial and ethnic identities, and socioeconomic statuses.

### Limited attention to treatment

Discussions of ED treatment in lecture slides were often limited to naming evidence-based therapeutic approaches or levels of care without further detail. This finding makes sense for preclinical education, as detailed training in treatment approaches is typically reserved for later stages of medical education. At the same time, it remains important that gaps in ED treatment education are addressed in subsequent phases of training, particularly during residency for those entering fields (e.g., child and adolescent psychiatry, pediatrics, adolescent medicine, family medicine) where EDs are routinely encountered. Indeed, research suggests that clinicians are less likely to diagnose conditions they feel unprepared to treat [31, 37], highlighting the need for future physicians to become well-versed in referral pathways to support timely assessment and intervention.

### Recommendations for enhancing preclinical medical education on EDs

This analysis suggests several changes to improve preclinical medical education on EDs:

1. Teach students to include EDs in their list of differential diagnoses when patients present with vague physical (e.g., weight loss, menstrual irregularity, fatigue, constipation, anemia, dizziness) or cognitive (e.g., poor concentration, insomnia) symptoms to support earlier detection of and intervention for EDs.
2. Ensure equitable inclusion of all EDs, including BED, ARFID, and OSFED, including how to identify malnutrition in non-emaciated bodies, to facilitate earlier detection and treatment.
3. Explicitly address weight stigma as both an ED risk factor and a barrier to care, emphasizing the ways healthcare professionals can mitigate weight stigma in clinical settings.
4. Ensure wide and diverse representation in images and vignettes of individuals experiencing EDs. For example, a slide of 20 individuals with diverse phenotypes who all have AN/atypical AN could be included to dismantle student’s preexisting biases.
5. Provide foundational information on assessment, treatment options, and levels of care, including how behavioral, social, cognitive, and/or physical symptoms may signal the need for a higher level of care, which can be deepened throughout training.

Improving ED education requires not only revising content but also fostering collaboration among medical educators, students, and ED specialists. Engaging these stakeholders in the development of lecture materials may help ensure that presentations are both evidence-based and aligned with the realities of medical training. A promising approach is the co-development of ED training modules that medical schools could integrate into preclinical curricula, incorporating expertise from physicians, researchers, patients, medical educators, and medical students. An online, centralized training module, modeled after existing efforts such as those developed for value-based health care [38], that is accessible to health professions at any stage of training and regularly updated could also facilitate the rapid integration of new research and treatment advancements, ensuring that ED education remains current. This approach could make ED education more inclusive, representative, accurate, and clinically relevant, ultimately improving early detection and treatment. Indeed, brief online training modules on EDs have been shown to increase healthcare professional’s confidence and competence in diagnosing and managing EDs [39, 40], making this strategy especially promising for preclinical medical education.

### Strengths and limits

This study assesses how EDs are portrayed in lecture presentations during preclinical medical education, a foundational period during which ED training has not previously been explored. A key strength is the analysis of both textual and visual content, which provides a more holistic understanding of implicit and explicit messages conveyed in training materials. Further, the use of directed qualitative content analysis, conducted by multiple coders, ensured a systematic approach. Limitations of the study include a relatively small sample of medical schools providing materials, a focus solely on lecture presentations, which does not account for other sources of knowledge about EDs in preclinical years (e.g., case-based learning or board exam preparation materials), and a focus only on slide content, meaning some gaps identified in the slides may have been supplemented with verbal commentary and discussion. Additionally, although we attempted to obtain materials from all. U.S. medical schools, only a subset provided lecture materials, which introduces potential sampling bias. Schools whose materials were unavailable may differ systematically from those included, such as in their curricular emphasis on EDs or the extent to which they have updated content in recent years. As a result, our findings may not fully represent the range of ways EDs are taught across U.S. medical schools. Finally, the courses in which these lectures were taught were not specified, and it is possible that relatively absent content (e.g., stigma) is covered in other parts of the curriculum.

### What is already known on this subject?

Physicians can play a critical role in the early identification and treatment of EDs, yet many report low confidence in diagnosing and managing these illnesses. Lack of comprehensive ED education in medical training may be a key contributor to this low confidence. Indeed, opportunities for training in EDs are limited, particularly at the residency level.

### What this study adds?

This study is the first to assess the content of ED training in preclinical medical education, highlighting gaps in how EDs are taught to medical students. Addressing these gaps is essential to improving physician’s abilities to identify and treat EDs across diverse patient populations. A crucial next step will be partnering medical educators with ED clinicians, medical students, researchers, and individuals with lived experience to develop evidence-based, inclusive, and clinically relevant educational materials for preclinical training.

## Data Availability

The data analyzed during the current study are available from the corresponding author on reasonable request.
